# Analysis of Antimicrobial Susceptibility in Bacterial Pathogens Associated with Urinary Tract Infections from Beijing Teaching Hospital in China, 2009–2017

**DOI:** 10.1155/2023/4360342

**Published:** 2023-07-24

**Authors:** Zeqiang Xie, Jiyong Jian, Liang Chen

**Affiliations:** ^1^Clinical Laboratory Medicine, Beijing Shijitan Hospital, Capital Medical University, Beijing, China; ^2^Peking University Ninth School of Clinical Medicine, Beijing, China; ^3^Beijing Key Laboratory of Urinary Cellular Molecular Diagnostics, Beijing, China

## Abstract

**Objective:**

Since a urinary tract infection (UTI) is easy to relapse and difficult to treat, the antibiotic resistance rate has increased year by year in recent years. This study was to analyze the characteristics of the common pathogenic bacteria and the changes of antibiotic resistance in urinary system infection, so as to guide the standard use of antibiotics in a clinical urinary tract infection and control nosocomial infection effectively.

**Methods:**

A total of 5,669 strains of a urinary tract infection in the hospital from January 2009 to December 2017 were retrospectively analyzed. Bacterial identification and the antibiotic sensitivity test (AST) were analyzed by using a VITEK-2 Compact system.

**Results:**

Of the 5669 pathogens, 3,256 (57.44%) of the strains were Gram-negative bacteria (GNB), 1,474 (26%) were Gram-positive bacteria (GPB), and 939 (16.56%) were fungi. Resistant rates of ESBL-producing strains were all significantly different from non-ESBL-producing strains in *Escherichia coli* (*p* < 0.05). The resistance rate of ESBL-producing strains to *β*-lactam antibiotics was all higher than that of non-ESBL-producing strains in *Klebsiella pneumoniae* (*p* < 0.05). The detection rate of vancomycin-resistant*Enterococcus faecium* and *Enterococcus faecalis* was 37.3% and 3.1%, respectively, and the detection rate of linezolid-resistant*Enterococcus faecium* and *Enterococcus faecalis* was 0.68% and 0%, respectively. The drug resistance rate of *candida* sp. to fluconazole, itraconazole, and voriconazole was 1.7%, 8.5%, and 3.4%, respectively. No amphotericin B-resistant strains were detected in the research.

**Conclusions:**

Among the 5669 strains isolated from urinary tract infection patients, GNB were the main pathogens. *Escherichia coli* was the major pathogen. The resistance rate of ESBLs-producing*Escherichia coli* was higher than that of non-ESBLs-producing*Escherichia coli* in general; meanwhile, *β*-lactam/*β*-lactamase inhibitors and carbapenems maintained good antimicrobial activity against *Escherichia coli*. The resistance rate of non-ESBLs-producing*Klebsiella pneumoniae* strains was significantly higher than that of ESBLs-producing*Klebsiella pneumoniae* strains, and drug resistance was more prominent; most of the antibiotic resistance rates were over 50%. The antimicrobial resistance rate of *Enterococcus faecium* was significantly higher than that of *Enterococcus faecalis*. There were rare linezolid-resistant strains. The antimicrobial resistance rate of imidazole to fungi was controlled less than 10%.

## 1. Introduction

A urinary tract infection (UTI) is one of the most common infectious diseases following the upper respiratory tract infection and is the main cause of morbidity and mortality in humans [1]. Pathogens can grow in the urinary tract and invade the urinary tract mucosa or tissue.

150 million people are infected with UTI each year worldwide [2]. A urine culture is currently the key standard for the diagnosis of a urinary tract infection [3]. A catheter-associated urinary tract infection is the most common nosocomial infection. With the prolongation of catheter placement time, the risk of urinary tract infections gradually increases. Some data indicate that the incidence of the urinary tract infection increases gradually with age, and most patients are bedridden due to underlying diseases such as cerebrovascular diseases, which is due to the frequent need for indwelling urinary catheters, thus significantly increasing the risk of urinary tract infections. *Escherichia coli* was the most frequent microbial agent of UT [4]. Uropathogenic *Escherichia coli* (UPEC) strains are the main pathogenic bacteria that cause urinary tract infections (UTIs), due to the presence of fimbrial adhesins in the pathogen [5]. One of the major difficulties with diagnosis of UTI in older people is a high prevalence of asymptomatic bacteriuria (ASB): the presence of bacteria in urine of people without attributable symptoms [6]. For this group of patients, monitoring urine cultures is an effective method to diagnose the disease. Urinary tract candidiasis is the most common hospital fungal infection worldwide, and this phenomenon cannot be ignored [7]. Effective drugs can help doctors treat urinary tract infections more effectively [8]. The abuse or overuse of antibiotics worldwide and antimicrobial resistance (AMR) are considered one of the top ten threats to global health [9]. For pathogens causing urinary tract infections, it is of great significance to analyze the infection characteristics for clinical diagnosis and treatment. The classification and analysis of urinary data for this hospital from 2009 to 2017 could help understand the distribution characteristics and trends of nosocomial pathogens and help doctors standardize drug use.

## 2. Materials and Methods

### 2.1. Bacterial Isolates

**Figure 1:**
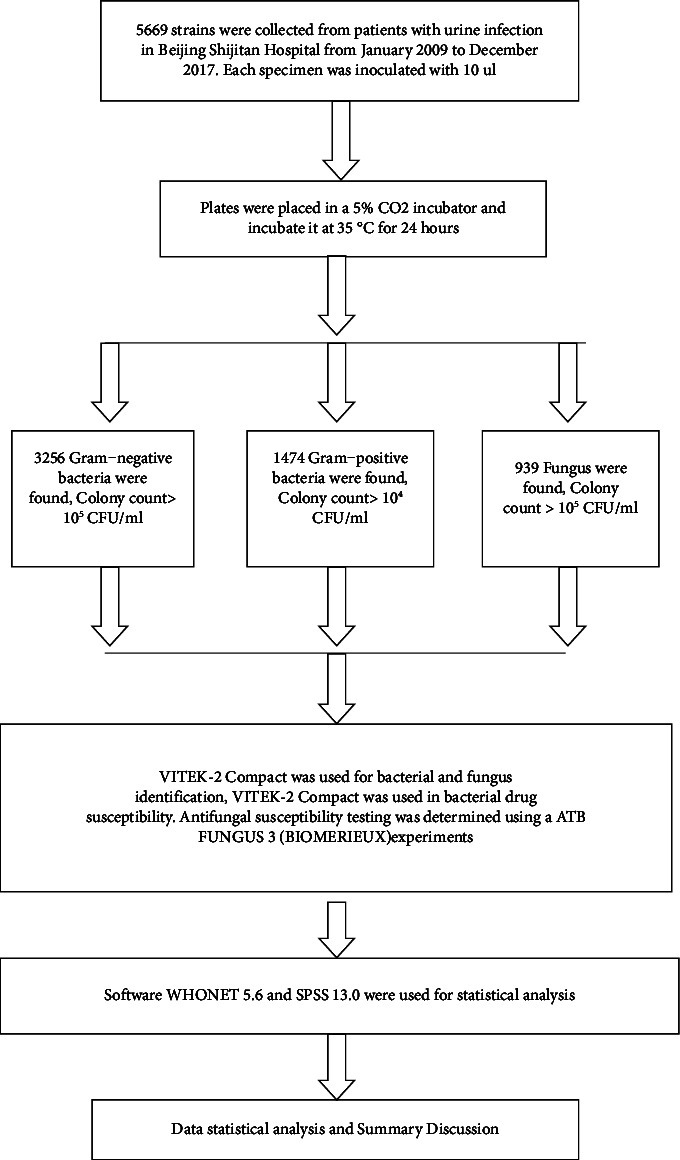
Flowchart of bacterial identification drug sensitivity operation procedure.

The clinical data on patients with urinary tract infections in Beijing Shijitan Hospital from January 2009 to December 2017 were collected. A total of 5,669 strains of pathogenic bacteria were isolated from urine samples. Strict aseptic procedures were carried out during specimen collection. Laboratory data requirements do not allow multiple isolates from the same patient to be counted twice. We take a 10 *μ*l urine sample and inoculate it tightly onto a Columbia blood plate. Plates were placed in a 5% CO_2_ incubator and incubated at 35°C for 24 hours. The number of bacterial colonies was counted on the blood plate (Figure 1).

The inclusion criteria for pathogens were as follows: 1. Gram-negative bacteria >10^5^ CFU/ml or Gram-positive bacteria >10^4^ CFU/ml or fungi >10^5^ CFU/ml. 2. The pathogenic bacteria were less than 3 pathogenic bacteria, otherwise considered pollution.

### 2.2. Antimicrobial Susceptibility Testing

The bacterial culture and the antibiotic susceptibility test (AST) were performed according to the National Guide to Clinical Laboratory Procedures (4^th^ edition). VITEK-2 Compact was used for bacteria land fungus identification. VITEK-2 Compact was used in bacterial drug susceptibility experiments. The bacterial's AST results were judged according to Performance Standards for Antimicrobial Susceptibility Testing M100 (31^st^ Edition). Antifungal susceptibility testing was determined using an ATB FUNGUS 3(BIOMERIEUX) strip following the manufacturer's instructions. Antifungal susceptibility testing results were judged according to Performance Standards for Antimicrobial Susceptibility Testing M60.

Ceftazidime/ceftazidime-clavulanate was used to validate the production of ESBLs for isolates according to CLSI. The ESBL-producing strain was judged to have increased ≥5 mm after the addition of clavulanic acid in the inhibition zone compared with the inhibition zone without clavulanic acid. *E coli* ATCC25922 and *K. pneumonia* ATCC 700603 were used as controls in the ESBL test.

### 2.3. Data Analysis

Software WHONET 5.6 and SPSS 13.0 were used for statistical analysis. Categorical variables were expressed as percentages and analyzed by using *χ*^2^. *p* < 0.05 was considered statistically significant.

## 3. Results

### 3.1. Overall Distribution of Urinary Pathogens

As shown in Table 1, a total of 5,669 pathogens were detected, 3,256 (57.44%) were Gram-negative bacteria (GNB), 1,474 (26%) were Gram-positive bacteria (GPB), and 939 (16.56%) were fungi. GNB presented the most prevalent part of the isolated pathogens. The two major isolates of GNB were *Escherichia coli* (26.37%) and *Klebsiella pneumoniae* (9.6%). The top two of GPB were *Enterococcus faecium* (12.93%) and *Enterococcus faecalis* (6.39%). The top three of fungi were *Candida albicans* (6.7%), *Candida glabrata* (4.22%), and *Candida tropical* (3.55%).

### 3.2. Gender Distribution of Pathogenic Bacteria in the Urinary System

During the past 9 years, the proportion of male and female urinary tract infections caused by pathogenic bacteria was 48.69% in female and 51.31% in male (Table 2).

### 3.3. Age Distribution of Pathogenic Bacteria in the Urinary System

As shown in Table 3, the top three age groups in urinary tract infections during the past 9 years were 81∼90 age group, 71∼80 age group, and 61∼70 age group, with 44.26%, 26.27%, and 10.23%, respectively. The population with urinary tract infections was mainly distributed among elderly people over 60 years old, with the highest proportion being those aged 81–90.

### 3.4. Susceptibility of *E.coli* to Antimicrobial Agents

There were 1495 *E.coli* isolates from the urinary system. It contained 651 ESBL-producing *E. coli* strains and 844 non-ESBL-producing *E. coli* strains, with a composition ratio of 43.55% and 56.45%, respectively. The drug resistance of ESBL-producing *E. coli* was higher than that of non-ESBL-producing*E. coli*, and the resistance rate was significantly different (*p* < 0.05). As shown in Table 4, ESBL-producing strains were more resistant to multiple drugs and had better effects on piperacillin/tazobactam and imipenem. The non-ESBL-producing strains had high resistance rates of ampicillin and quinolones.

### 3.5. Susceptibility of *Klebsiella pneumoniae* to Antimicrobial Agents

As shown in Table 5, a total of 544 strains of *Klebsiella pneumoniae* were isolated from the urinary system, including 126 strains of ESBL-producing *Klebsiella pneumoniae* and 418 non-ESBL-producing strains of *Klebsiella pneumoniae*, with a composition ratio of 23.16% and 76.84%, respectively. Non-ESBL-producing strains were significantly more than ESBL-producing strains in the resistance rate of *β*-lactam drugs (*p* < 0.05), whereas the drug resistant rate of aminoglycosides, quinolones, and furans showed no significant difference (*p* < 0.05). ESBL-producing strains were highly resistant to most *β*-lactams. Piperacillin/tazobactam and imipenem had high resistance rates and better drug sensitivity. For non-ESBL-producing strains, the form of imipenem resistance was not optimistic, and most of the beta-lactam drugs were not effective. The resistance rate of quinolone drugs and furan drugs was higher.

### 3.6. Susceptibility of the Gram-Positive Bacteria

As shown in Table 6, the Gram-positive bacteria of urinary tract infections mainly consisted of *Enterococcus faecium* and *Enterococcus faecalis*. Among them, 362 strains of *Enterococcus faecalis* and 733 strains of *Enterococcus faecium* had a composition ratio of 33.06% and 66.94%, respectively. *Enterococcus faecium* was significantly more resistant than *Enterococcus faecalis*. Linezolid-resistant strains were not found, the VRE detection rate of *Enterococcus faecalis* was below 10%, and the quinolone resistance rate fluctuated around 50% in *Enterococcus faecalis*.

The resistance rate of *Enterococcus faecium* to ampicillin was higher, the resistance rate of ciprofloxacin and erythromycin was higher, clinically linezolid-resistant strains were few, and the resistance rate of vancomycin-resistant *Enterococcus faecium* was 37.3%; meanwhile, the resistance rate of quinupristin/davapride was 3.9%.

### 3.7. Susceptibility of Fungi to Antimicrobial Agents

Amphotericin *B*-resistant strains were not found among the main yeasts in urinary tract infections. The average resistance rate of the imidazole antifungal drug was below 10% (Table 7).

## 4. Discussion

This article makes statistics on pathogens of urinary tract infections in our hospital and provides reasonable and effective data support for clinicians in the treatment of diseases. The existence of resistance mechanisms such as *β*-lactamases, efflux pumps, membrane permeability, and change in the sequence of penicillin-binding proteins (PBPs) in bacteria has made the treatment of multidrug-resistant bacteria a very challenging issue globally [10]. The relationship between antimicrobial phenotypes and biofilm formation ability has always been a topic of widespread concern in the biomedical community. Scientists believe that these two factors may have a significant impact on the outcome of infection [11]. Studies have shown that a novel biofilm preventive agent (BPA) can inhibit the formation of biofilms by pathogens on the catheter [12].

ESBL-producing *Escherichia coli* isolates and ESBL-negative isolates had significant differences in drug resistance rates (*p* < 0.05) in ampicillin, piperacillin/tazobactam, ceftazidime, aztreonam, imipenem, amikacin, ciprofloxacin, levofloxacin, and so on. The drug resistance rate of ESBL-producing *Escherichia coli* isolates was significantly higher than that of ESBL-negative isolates. Imipenem and piperacillin/tazobactam maintained higher antibacterial activity. No imipenem-resistant strain was found in EBSL-producing strains. The resistance rate of *Escherichia coli* to amikacin was the lowest among aminoglycoside drugs. Aminoglycoside antibiotics mainly act on G-bacilli and have strong and long-lasting antibacterial activity. However, due to its toxicity to the ear and kidney, its usage rate is decreasing year by year. Comprehensive evaluation of renal function is recommended for patients with urinary tract infections. The resistance rate of *Escherichia coli* to quinolones was high, and drug resistance situation was not optimistic. The drug is widely used due to the wide antimicrobial spectrum of quinolones. The resistance mechanism of quinolone is the carrier of quinolone-resistant genes, such as qnr (qnrA, qnrB, and qnrS), aac(6′)-Ib-cr, qepA, and oqxAB [13]. It required further study. At present, quinolones are not recommended to use. Uropathogenic *Escherichia coli* (UPEC) is the most important bacteria causing urinary tract infections (UTIs) in patients worldwide. Scholars have proved that DNA microarray was a suitable diagnostic technique for detecting and identifying diseases in UPEC [14]. It has been suggested to design a suitable long oligonucleotide microarray probe for the detection and identification of different pathogenic *Escherichia coli*, especially urinary pathogenic *Escherichia coli* (UPEC) [15]. A UPEC pathogen is considered to be an important microorganism with multiple virulence genes. Understanding the bacterial virulence gene map and related functional mechanisms enables us to design effective preventive approaches for the treatment of urinary tract infections [16]. The study from Iran showed that cnf1, upaH, hlyA, ibeA, and cdtB were important virulence genes in UPEC pathogens isolated from urine. The virulence gene of cnf1 in UPEC strains contributes to the occurrence of various types of urinary tract infections [17].

The resistance rates of ESBL-producing *Klebsiella pneumonia* isolates to piperacillin/tazobactam, cefazolin, aztreonam, and imipenem were significantly different from those of ESBL-negative isolates (*p* < 0.05). The drug resistance rates were not significantly different (*p* < 0.05) in amikacin and ciprofloxacin. The current statistical results indicated that the resistance rate of ESBL-negative isolates strains was significantly higher than that of ESBL-producing *Klebsiella pneumonia*. The resistance rate of ESBL-producing*Klebsiella pneumonia* to piperacillin/tazobactam was 13.5%; the result was similar to the previous report [18]. While the drug resistance rate to imipenem was only 2.4%, the main resistance mechanism was carbapenemase.

The first carbapenemase-producing *Klebsiella pneumoniae* was first reported in the United States in 2001 [19]. The KPC-type carbapenemase producing *Klebsiella pneumoniae* has rapidly spread around the world, and outbreaks have been reported in hospitals around the world. The resistance rate of *β*-lactam antibiotics was on the rise. The rate of drug resistance was similar to those reported in many other countries [20, 21]. The resistance of *Klebsiella bacteria* to carbapenem antibiotics is mainly caused by carbapenemase production (such as production of class A KPC enzymes and class B MBL enzymes). Carbapenem-resistant *Klebsiella pneumoniae* (CRKP) infection poses a major threat to public health. Understanding the exact mechanism by which CRKP occurs is crucial for selecting the most appropriate antibiotic [22]. Strains may or may not have resistance mechanisms such as ESBL, AmpC *β*-lactamase, efflux pumps, and membrane pore protein mutations [23, 24]. Currently, multidrug-resistant bacteria require combination drug treatment, such as tigecycline-based two-drug combination: tigecycline + carbapenems/polymyxins/aminoglycosides or polymyxin-based combination of drugs: polymyxins + carbapenems/fosfomycin/aminoglycosides or three-drug combinations: tigecycline + carbapenems + polymyxins [25].

As shown in Table 6, the drug-resistant rate of *Enterococcus faecium* was significantly higher than that of *Enterococcus faecalis*. The resistant rate of vancomycin-resistant *Enterococcus faecium* was 37.3%, which was significantly higher than in foreign reports. Since the first report of vancomycin-resistant enterococci (VRE) in Britain [26] and France [27] in 1988, VRE has become an important drug-resistant strain that seriously threatens human health, and it has caused great difficulties for clinical treatment. According to the US CDC report, at least 20,000 cases of medically associated infections caused by VRE occurred each year, and nearly 1,300 died of VRE infection [28]. The possible causes may be related to the production of resistance genes such as VanA, VanB, VanC, and VanD [29]. The fluctuation of the VRE detection rate of vancomycin-resistant *Enterococcus faecium* was higher than that in the US [30], the Netherlands, and Germany [31]. Five strains of linezolid-resistant enterococci were isolated. Although these strains are rare, their resistance mechanism may be related to G2576T point mutation in the 23S rDNA gene and plasmid-mediated resistance of the cfr gene [32]. The specific mechanism requires further study. *Enterococcus faecalis* was significantly more sensitive than *Enterococcus faecium*. The resistance rate of vancomycin was 3.1%, and no linezolid-resistant strains were found. Quinolone drugs have a high resistance rate in the treatment of urinary tract infections.

The high rate of resistance to quinolones in the treatment of urinary tract infections is an indisputable fact. Hospital management departments must strictly control and take necessary measures to curb its high-drug resistance.

In urinary tract infections, *Candida* spp. are the most common fungal cause of urinary tract fungal infections [33]. Among the patients with urinary system fungal infections, 469 were male patients and 470 were female patients, which was basically the same. No amphotericin *B*-resistant strain is found in any of the three major yeasts in Table 7. This is also associated with adverse effects of amphotericin *B*, which can cause hypokalemia, renal damage, and liver damage. The resistance rate of fluconazole was the lowest (1.7%). Fluconazole has high efficacy, low toxicity, and mild adverse reactions. It is excreted by the kidney, and the concentration in urine is 10 times that of blood, so it is the first choice for urinary tract fungal infections. Voriconazole has high drug sensitivity. It is suitable for urinary tract infections caused by fungi.

## 5. Conclusions

Among 5,669 strains isolated from patients with urinary tract infections, GNB were the main pathogens. The drug resistance rate of ESBL-producing *E. coli* was higher than that of ESBL-negative *E. coli*. in general. The resistant rate of ESBL-negative *Klebsiella pneumoniae* was significantly higher than that of ESBL-producing *Klebsiella pneumoniae*. The resistance rate of *Enterococcus faecium* to various antibacterial drugs was significantly higher than that of *Enterococcus faecalis*. The results of the urine culture are the basis of clinical treatment. Clinicians should keep abreast of the changes in drug resistance of pathogens in hospitals. Rational use of antibiotics is very important in clinical diagnosis and treatment.

## Figures and Tables

**Table 1:** Distribution and constituent ratio of pathogens in patients with a urinary tract infection (%).

Pathogenic bacteria	No. of isolates	Composition ratio	Female	Male	*χ* ^2^	*p*
**Gram-negative bacteria**	**3,256**	**57.44**	**1,524**	**1,732**	**10.821**	*p* < 0.05
*Escherichia coli*	1,495	26.37	431	1,064	320.447	*p* < 0.05
*Klebsiella pneumoniae*	544	9.6	280	264	1.868	*p* > 0.05
*Pseudomonas aeruginosa*	400	7.06	287	113	91.638	*p* < 0.05
*Proteus mirabilis*	230	4.06	150	80	26.224	*p* < 0.05
*Acinetobacter baumannii*	207	3.65	157	50	63.435	*p* < 0.05
*Klebsiella oxytoca*	58	1.02	23	35	1.913	*p* > 0.05
*Enterobacter cloacae*	55	0.97	27	28	0.004	*p* > 0.05
*Morganella morganii*	45	0.79	26	19	1.501	*p* > 0.05
*Stenotrophomonas maltophilia*	31	0.55	26	5	15.446	*p* < 0.05
*Citrobacter freundii*	30	0.53	16	14	0.261	*p* > 0.05
Other	161	2.84	101	60	13.088	*p* < 0.05
**Gram-positive bacteria**	**1,474**	**26**	**767**	707	8.945	*p* < 0.05
*Enterococcus faecium*	733	12.93	353	380	0.094	*p* > 0.05
*Enterococcus faecalis*	362	6.39	212	150	15.102	*p* < 0.05
*Staphylococcus epidermis*	80	1.41	53	27	10.02	*p* < 0.05
*Staphylococcus aureus*	66	1.16	44	22	8.642	*p* < 0.05
*Enterococcus gallinarum*	43	0.76	22	21	0.106	*p* > 0.05
*Enterococcus casseliflavus*	27	0.48	8	19	3.943	*p* < 0.05
*Streptococcus agalactiae*	25	0.44	6	19	6.125	*p* < 0.05
Other	138	2.43	69	69	0.098	*p* > 0.05
**Fungi**	**939**	**16.56**	**469**	470	0.716	*p* > 0.05
*Candida albicans*	380	6.7	179	201	0.407	*p* > 0.05
*Candida glabrata*	238	4.2	87	151	14.634	*p* < 0.05
*Candida tropicalis*	201	3.55	119	82	9.228	*p* < 0.05
*Candida parapsilosis*	68	1.2	55	13	28.558	*p* < 0.05
Other	52	0.91	29	23	1.054	*p* > 0.05
**Total**	**5,669**	**100**	**2,760**	**2,909**		

The bold values are the sum of the classified and summarized data, indicating emphasis.

**Table 2:** Gender distribution of pathogenic bacteria in the urinary system (%).

Gender distribution	No. of isolates	Composition ratio (%)
Female	2,760	48.69
Male	2,909	51.31
Total	5,669	100.00

**Table 3:** Age distribution proportion of patients with urinary tract infections (%).

Age	No. of isolates	Composition ratio (%)
1–10	9	0.16
11–20	12	0.21
21–30	67	1.18
31–40	66	1.16
41–50	155	2.73
51–60	354	6.24
61–70	580	10.23
71–80	1,489	26.27
81–90	2,509	44.26
>90	428	7.55
Total	5,669	100.00

**Table 4:** Susceptibility of *E.coli* to antibiotics.

Antibacterial drugs	ESBL (+) strains (*n* = 651)		ESBL (−) strains (*n* = 844)		*χ * ^2^	*p*
	No. of isolates	*R*	No. of isolates	*R*		
Ampicillin	648	99.5	594	70.4	222.28	*p* < 0.05
Cefazolin	627	96.3	174	20.6	846.71	*p* < 0.05
Ciprofloxacin	545	83.7	495	58.7	109.08	*p* < 0.05
Levofloxacin	522	80.2	464	55	104	*p* < 0.05
Ceftriaxone	511	78.5	117	13.9	630.21	*p* < 0.05
Ampicillin/sulbactam	473	72.6	257	30.5	262.03	*p* < 0.05
Trimethoprim-sulfamethoxazole	388	59.6	404	47.9	20.31	*p* < 0.05
Gentamicin	387	59.4	377	44.7	32.12	*p* < 0.05
Aztreonam	221	33.9	88	10.4	124.01	*p* < 0.05
Tobramycin	184	28.3	112	13.3	52.04	*p* < 0.05
Cefepime	175	26.9	78	9.2	81.34	*p* < 0.05
Ceftazidime	90	13.8	68	8.1	12.94	*p* < 0.05
Amikacin	39	6	20	2.4	12.71	*p* < 0.05
Nitrofurantoin	36	5.6	46	5.4	0.01	*p* > 0.05
Cefotetan	8	1.3	49	5.8	20.99	*p* < 0.05
Piperacillin/tazobactam	7	1.1	52	6.2	25.08	*p* < 0.05
Imipenem	0	0	30	3.6	23.61	*p* < 0.05

**Table 5:** Susceptibility of *K. pneumoniae* to antibiotics.

Antibacterial drugs	ESBL (+) strains (*n* = 126)		ESBL (−) strains (*n* = 418)		*x * ^2^	*p*
	No. of isolates	*R*	No. of isolates	*R*		
Cefazolin	124	98.4	286	68.5	46.91	*p* < 0.05
Ampicillin/sulbactam	109	86.7	301	71.9	10.96	*p* < 0.05
Trimethoprim-sulfamethoxazole	97	76.9	187	44.7	40.35	*p* < 0.05
Ceftriaxone	89	70.7	252	60.2	4.43	*p* < 0.05
Gentamicin	89	70.6	261	62.4	2.83	*p* > 0.05
Nitrofurantoin	81	63.9	284	68	0.59	*p* > 0.05
Ciprofloxacin	78	62.1	273	65.2	0.49	*p* > 0.05
Levofloxacin	73	57.7	261	62.5	0.83	*p* > 0.05
Aztreonam	66	52	264	63.1	4.71	*p* < 0.05
Tobramycin	56	44.7	193	46.2	0.12	*p* > 0.05
Amikacin	39	30.9	147	35.1	0.76	*p* > 0.05
Cefepime	38	30.2	229	54.7	23.49	*p* < 0.05
Ceftazidime	33	26	231	55.3	32.76	*p* < 0.05
Piperacillin/tazobactam	17	13.5	257	61.4	89.20	*p* < 0.05
Cefotetan	10	7.6	227	54.3	84.67	*p* < 0.05
Imipenem	3	2.4	235	56.3	114.04	*p* < 0.05

**Table 6:** Susceptibility of *Enterococcus faecalis* and *Enterococcus faecium* to antibiotics.

Antibacterial drugs	*Enterococcus faecalis* (*n* = 362)		*Enterococcus faecium* (*n* = 733)		*X * ^2^	*p*
	No. of isolates	*R*	No. of isolates	*R*		
Tetracycline	314	86.7	238	32.5	285.51	*p* < 0.05
Erythromycin	282	78	685	93.4	56.77	*p* < 0.05
Ciprofloxacin	195	54	726	99.1	370.05	*p* < 0.05
High concentrations of gentamicin	77	21.4	181	24.7	1.57	*p* > 0.05
Vancomycin	11	3.1	273	37.3	147.6	*p* < 0.05
Ampicillin	7	1.8	726	99.1	1032.65	*p* < 0.05
Linezolid	0	0	5	0.68	1.21	*p* > 0.05
Quinupristin/ davapride	NA	NA	29	3.9	—	—

**Table 7:** Susceptibility of antifungal agents.

Antibacterial drugs	*Candida albicans (n* *=* 380)		*Candida glabrata* (*n* = 238)		*Candida tropicalis* (*n* = 201)		*X* ^2^	*p*
	No. of isolates	*R* (%)	No. of isolates	*R* (%)	No. of isolates	*R* (%)		
Amphotericin *B*	0	0	0	0	0	0	—	—
Fluconazole	0	5	5	2.1	10	5	18.233	*p* < 0.05
Itraconazole	13	3.4	40	16.8	14	7	35.432	*p* < 0.05
Voriconazole	8	2.1	7	2.9	16	8	13.031	*p* < 0.05

## Data Availability

The data used to support the findings of this study are included within the article.
